# Determinants of Chromosome Architecture: Insulator Pairing in *cis* and in *trans*

**DOI:** 10.1371/journal.pgen.1005889

**Published:** 2016-02-24

**Authors:** Miki Fujioka, Hemlata Mistry, Paul Schedl, James B. Jaynes

**Affiliations:** 1 Deptartment of Biochemistry and Molecular Biology, Thomas Jefferson University, Philadelphia, Pennsylvania, United States of America; 2 Departments of Biology and Biochemistry, Widener University, Chester, Pennsylvania, United States of America; 3 Department of Molecular Biology, Princeton University, Princeton, New Jersey, United States of America; 4 Laboratory of Gene Expression Regulation in Development, Institute of Gene Biology, Russian Academy of Sciences, Moscow, Russia; Netherlands Cancer Institute, NETHERLANDS

## Abstract

The chromosomes of multicellular animals are organized into a series of topologically independent looped domains. This domain organization is critical for the proper utilization and propagation of the genetic information encoded by the chromosome. A special set of architectural elements, called boundaries or insulators, are responsible both for subdividing the chromatin into discrete domains and for determining the topological organization of these domains. Central to the architectural functions of insulators are homologous and heterologous insulator:insulator pairing interactions. The former (pairing between copies of the same insulator) dictates the process of homolog alignment and pairing in *trans*, while the latter (pairing between different insulators) defines the topology of looped domains in *cis*. To elucidate the principles governing these architectural functions, we use two insulators, Homie and Nhomie, that flank the *Drosophila even skipped* locus. We show that homologous insulator interactions in *trans*, between Homie on one homolog and Homie on the other, or between Nhomie on one homolog and Nhomie on the other, mediate transvection. Critically, these homologous insulator:insulator interactions are orientation-dependent. Consistent with a role in the alignment and pairing of homologs, self-pairing in *trans* is head-to-head. Head-to-head self-interactions in *cis* have been reported for other fly insulators, suggesting that this is a general principle of self-pairing. Homie and Nhomie not only pair with themselves, but with each other. Heterologous Homie-Nhomie interactions occur in *cis*, and we show that they serve to delimit a looped chromosomal domain that contains the *even skipped* transcription unit and its associated enhancers. The topology of this loop is defined by the heterologous pairing properties of Homie and Nhomie. Instead of being head-to-head, which would generate a circular loop, Homie-Nhomie pairing is head-to-tail. Head-to-tail pairing in *cis* generates a stem-loop, a configuration much like that observed in classical lampbrush chromosomes. These pairing principles provide a mechanistic underpinning for the observed topologies within and between chromosomes.

## Author Summary

The chromosomes of multicellular animals are organized into a series of topologically independent looped domains. This domain organization is critical for the proper utilization and propagation of the genetic information encoded by the chromosome. A special set of architectural elements, called boundaries or insulators, are responsible for both subdividing the chromatin fiber into discrete domains, and determining the topological organization of these domains. Central to the architectural functions of insulators are heterologous and homologous insulator:insulator pairing interactions. In *Drosophila*, the former defines the topology of individual looped domains in *cis*, while the latter dictates the process of homolog alignment and pairing in *trans*. Here we use two insulators from the *even skipped* locus to elucidate the principles governing these two architectural functions. These principles align with several longstanding observations, and resolve a number of conundrums regarding chromosome topology and function.

## Introduction

The highly regular and reproducible physical organization of chromosomes in multicellular eukaryotes was recognized a century ago in cytological studies on the lampbrush chromosomes that are found in oocytes arrested at the diplotene phase of meiosis I [1–3]. At this stage, homologous chromosomes are paired. The two homologs display a similar and reproducible architecture. It consists of a series of loops emanating from the main axis, that are arranged in pairs, one from each homolog. In between the loops are regions of more compacted chromatin [2]. A similar physical organization is evident in insect polytene chromosomes [4]. As with lampbrush chromosomes, the paired homologs are aligned in precise register. However, instead of one copy of each homolog, there are hundreds. While loops are not readily visible, each polytene segment has a unique pattern of bands and interbands that depends upon the underlying DNA sequence and chromosome structure.

Subsequent studies have shown that the key features of chromosome architecture evident in lampbrush and polytene chromosomes are also found in diploid somatic cells [5–13]. One of these is the subdivision of the chromatin fiber into a series of loop domains. There are now many lines of evidence indicating that looping is a characteristic architectural feature. Biochemical evidence comes from chromosome conformation capture (3C) experiments, which show that distant sites come into contact with each other in a consistent pattern of topologically associating domains (TADs). While the first studies in mammals suggested that TADs have an average length of 1 Mb [14–16], subsequent experiments showed that the average is only about 180 kb [17]. In flies, TADs are smaller, between 10–100 kb [18,19]. Neighboring TADs are separated from each other by boundaries that constrain both physical and regulatory interactions. In mammals and also in flies, these boundaries typically correspond to sequences bound by insulator proteins like CTCF [17].

That TAD boundaries correspond to insulators is consistent with the known properties of these elements. Insulators subdivide the chromosome into functionally autonomous regulatory domains. When interposed between enhancers or silencers and target promoters, insulators block regulatory interactions. They also have an architectural function in that they can bring distant chromosomal sequences together, and in the proper configuration can promote rather than restrict regulatory interactions [20,21]. Moreover, insulators are known to mediate contacts between distant sequences (loop formation), and these physical contacts depend upon specific interactions between proteins bound to the insulators [22,23].

The notion that insulators are responsible for subdividing eukaryotic chromosomes into a series of looped domains raises questions about the rules governing loop formation in *cis*. One of these is the basis for partner choice. Is choice based simply on proximity, or is there an intrinsic partner preference? A second concerns the topology of the loop formed by interacting partners in *cis*. Do the partners interact to form a stem-loop-like structure, or does the interaction generate a circular loop (“circle-loop”)? The answer to this question will depend upon whether there is an orientation dependence to the interactions between two heterologous insulators. In flies, homologs are typically paired in somatic cells, not just in cells that are polyploid [24]. This means that the loop domains in each homolog must be aligned in precise register along their entire length. A plausible hypothesis is that both alignment and homolog pairing are mediated by insulator interactions in *trans*. If this is case, there are similar questions about the rules that govern *trans* interactions. Is there a partner preference in the interactions that mediate homolog pairing? Is there an orientation dependence, and if so, what is the topological outcome of the looped domains generated by insulator interactions in paired chromosomes in *cis* and in *trans*?

In the studies reported here, we have used insulators from the *even skipped* (*eve*) locus to address the questions posed above about the architecture of eukaryotic chromosomes. The *eve* domain spans 16 kb and is bordered upstream by the Nhomie (Neighbor of Homie, this study) insulator and downstream by Homie (Homing insulator at *eve*) [25,26]. *eve* encodes a homeodomain transcription factor that is required initially for segmentation, and subsequently in the development of the CNS, muscles, and anal plate [27,28]. It has a complex set of enhancers that activate expression at different stages and tissues [25,29–31], and a Polycomb response element (PRE) that silences the gene in cells where it isn’t needed [32]. In early embryos, the stripe enhancers upstream (3+7, 2, late stripes) and downstream (4+6, 1, and 5) of the *eve* gene activate transcription in a pair-rule pattern. Later in development, around the time that germband retraction commences, mesodermal (Me) and neuronal (CNS) enhancers turn on *eve* expression in a subset of cells in each of these tissues. These late enhancers continue to function once germband retraction is complete, while another enhancer (APR) induces transcription in the presumptive anal plate. Located just upstream of *eve* is *CG12134*, while the *TER94* gene is downstream. Unlike *eve*, both of these genes are ubiquitously expressed throughout much of embryogenesis.

## Results

### Homie pairs with itself and with Nhomie

The Homie insulator has two striking properties [26]. First it directs homing of otherwise randomly inserting transgenes to a ~5 Mb region centered on the *eve* locus. Second, when the homed transgene carries a reporter, it is expressed in an *eve*-like pattern, the completeness of which diminishes with distance. Early stripe and later CNS expression are limited to 200 kb from *eve*, mesodermal expression has an intermediate distance dependence, while anal plate ring (APR) expression is seen at distances of several Mb. We showed previously that reporter expression at a site within the *hebe* gene 142 kb upstream of *eve* requires Homie [26]. Since other fly insulators mediate long-distance regulatory interactions by direct physical contact [22,33], we used high-resolution chromosome conformation capture (H3C) [34] to map contacts between transgenes at -142 kb and *eve* (see below).

**Fig 1:**
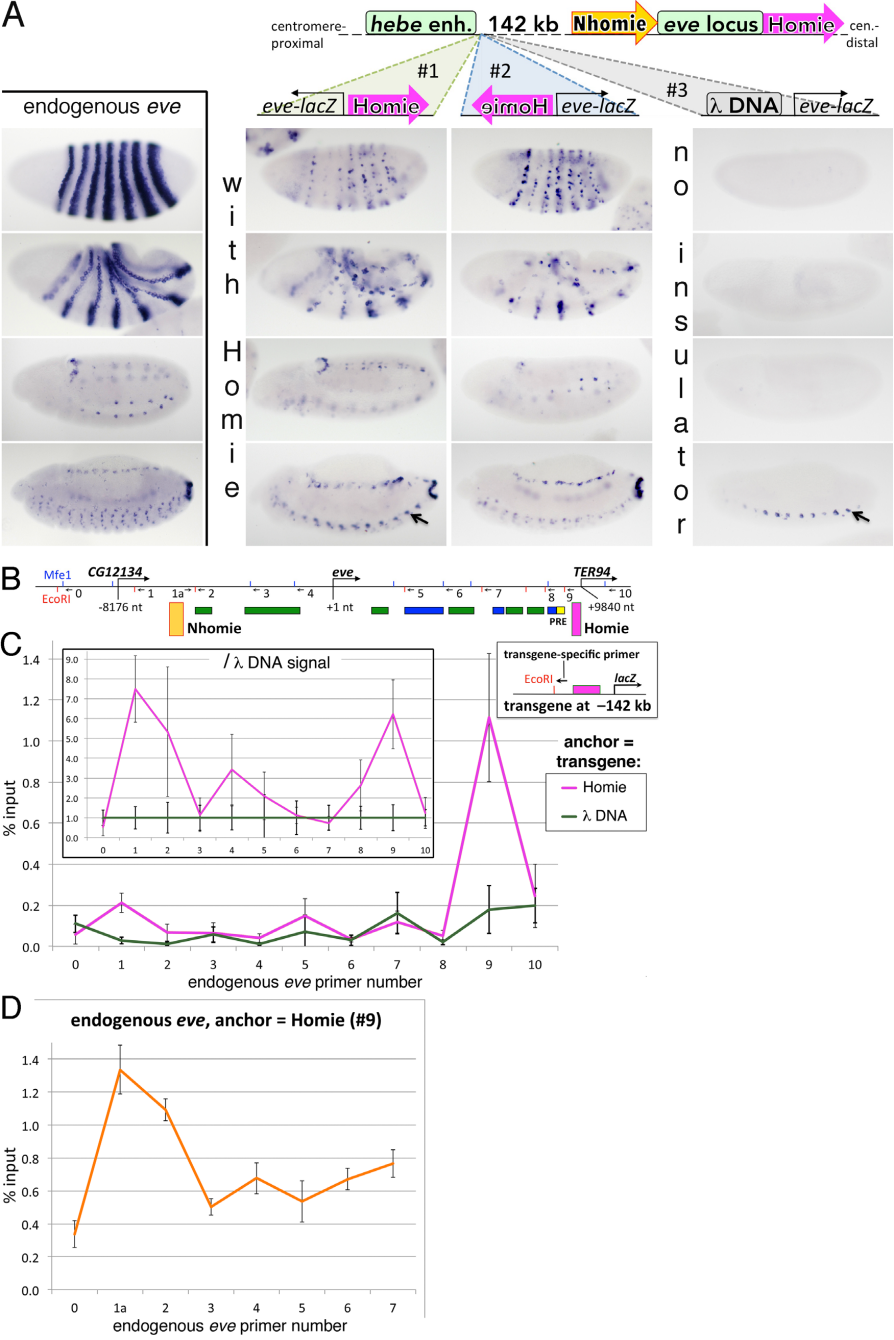
Homie-carrying transgenes pair with endogenous *eve*. **A:** *eve*-patterned *lacZ* RNA expressed from reporters with Homie is independent of transgene orientation. Embryonic stages 5–6, 7–8, 11, and 13 (top to bottom in each column) are shown. **Top:** diagram of chromosome (“*hebe* enh.”: *hebe* CNS enhancer; block arrows indicate the orientations of the insulators) and transgenes (#1–3) inserted at a common site located 142 kb from the *eve* locus. **1**^**st**^ **column**: endogenous *eve* RNA. **2**^**nd**^ **and 3**^**rd**^ **columns**: *lacZ* RNA; note that the whole transgene is oriented in opposite directions. Black arrow points to a *hebe* CNS pattern element that is repeated in each segment (bottom panel for transgenes #1 and #3), which is distinct from the *eve* CNS pattern that is also present, but in smaller cells; this *eve* pattern is seen in the bottom panel for transgene #2, where there is no *hebe*-like expression. **Right column**: control transgene #3; only the *hebe* CNS pattern is seen. **B:** Map of *eve* and flanking genes: restriction sites are shown as blue ticks (MfeI) and red ticks (EcoRI). Numbered arrows are primers used for H3C in C and D. Colored rectangles show the locations of stripe enhancers (green), later-acting enhancers (blue), a PRE (bright yellow) and insulators Homie (magenta) and Nhomie (yellow). **C:** Endogenous Homie and Nhomie link up with transgene Homie from 142 kb away. Inset map: locations of Homie (magenta box), replaced by DNA in the control, the transgene-specific anchor primer, and EcoRI site are shown. The main graph shows averages and standard deviations of 6 independent H3C assays, with each quantified by qPCR either in duplicate or in triplicate. The standard deviations of these qPCRs were aggregated as non-overlapping subsets of a parent set using a standard sample-based statistics formula. Colored lines connect data points for either the Homie-carrying transgene (magenta), or the DNA-carrying transgene (green). The data are presented as % input, which is ligated product representing an interaction between the transgene (anchor primer, common to all reactions) and a part of endogenous *eve* (numbered primer), divided by the total anchor fragment, separately quantified within each sample, as described in [34]. Inset graph: the same data normalized to the average signal with DNA in the transgene in place of Homie. **D:** Endogenous Homie and Nhomie interact with each other. Results of H3C, performed and presented as in C, except with primer 9 (at endogenous Homie) as the anchor.

The transgenes have an *eve*-promoter-*lacZ* (*lacZ*) reporter and Homie. One of them is inserted into the chromosome so that Homie is oriented in the same direction (→; Fig 1A, transgene #1) as the endogenous Homie in the *eve* locus, while the other transgene is inserted in the opposite orientation (←; Fig 1A, transgene #2). In the control transgene, Homie was replaced by DNA (Fig 1A, transgene #3). Fig 1A shows that the reporters in both Homie transgenes are regulated by the *eve* enhancers in a pattern which recapitulates that of endogenous *eve*. Thus, the orientation of the entire Homie:*lacZ* transgene in the chromosome doesn’t affect long-distance regulation. On the other hand, because of a *hebe* CNS enhancer located upstream of -142 kb, the expression pattern is not identical. In the transgene that is oriented so that Homie is closer to the *eve* locus than the reporter (Fig 1A, 2^nd^ column: transgene #1), *lacZ* is regulated by both the *hebe* CNS enhancer (arrow in bottom panel) and the *eve* enhancers (all 4 panels). When the transgene is inserted in the opposite orientation so that the *lacZ* reporter is closer to the *eve* locus (Fig 1A, 3^rd^ column: transgene #2), Homie blocks the *hebe* enhancer, and only the *eve* pattern is seen (all 4 panels). Finally, as expected, the reporter in the DNA control transgene (Fig 1A, right column: transgene #3) is not regulated by the *eve* enhancers (all 4 panels), but is regulated by the *hebe* enhancer (arrow in bottom panel). In this case, the reporter is separated from the *hebe* enhancer by DNA, not Homie. These results show that Homie induces a long-range interaction between a reporter transgene located many kilobases away and endogenous *eve* enhancers, and that this interaction is not sensitive to the orientation of the transgene in the chromosome. (However, this experiment does not test the orientation dependence of the reporter relative to the insulator, as this does not change between these two cases. This is tested below.) Furthermore, the long-range looping interactions between the transgene and the *eve* locus do not change the local enhancer blocking activity of the Homie insulator.

Since insulator bypass assays show that fly insulators pair with themselves [35–37], we expected that Homie:Homie pairing is responsible for long-distance regulation. However, as the transgene Homie might also interact with other *eve* elements, we used 11 primers spanning the locus (Fig 1B, arrows numbered 0–10) for H3C. Fig 1C shows the 3C results for the experimental and for the control DNA transgene, while in the inset we controlled for “non-specific” interactions using data from the DNA transgene as the reference. Whereas there is little interaction between the control transgene and the *eve* locus (Fig 1C green line), the experimental transgene shows significant interactions with two elements in the locus (magenta line). One is endogenous Homie. The other is located at the 5’ boundary of the *eve* Polycomb domain [38,39], and, from genome-wide chromatin immunoprecipitation studies [40], is bound *in vivo* by many insulator proteins. Based on these and findings below, we call this *eve* 5’ insulator Nhomie.

### Long-distance *regulatory* interactions are orientation dependent

**Fig 2:**
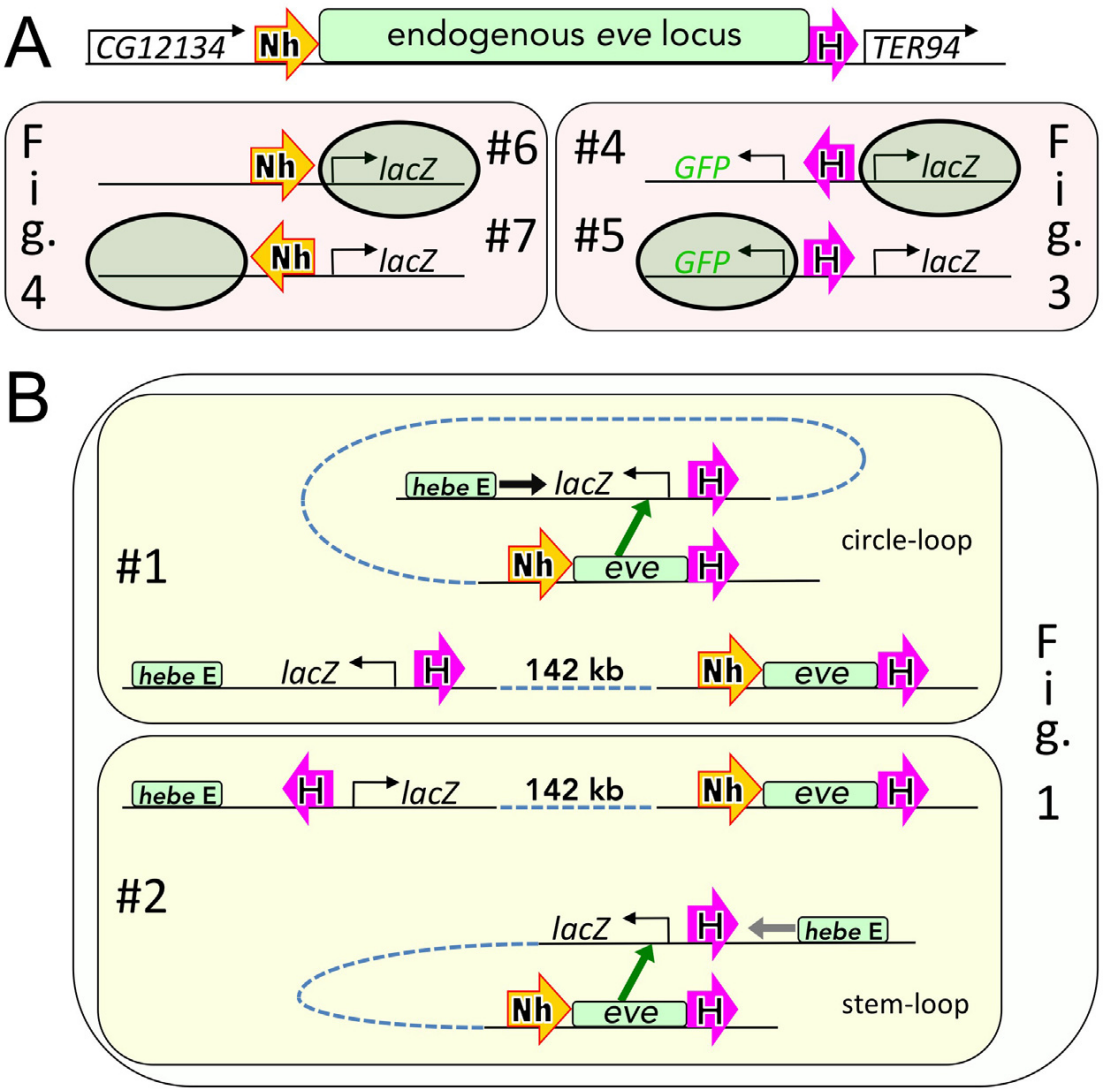
Diagrammatic comparison of insulator-containing transgenes (“#1”-“#7”) used in Figs 1, 3 and 4 (as indicated). Large block arrows show insulators and their orientations, as in Fig 1. **A:** locations and orientations of insulators and reporters in transgenes used in Figs 3 and 4 relative to the endogenous *eve* locus. In each transgene, the shaded oval marks the reporter that is in the same position relative to the insulator (Homie, “H”, or Nhomie, “Nh”) as are the endogenous *eve* enhancers (within the “endogenous *eve* locus”). **B:** locations of transgenes used in Fig 1 in the chromosome relative to endogenous *eve* (middle, linear diagrams) and topologies (“circle-loop” for transgene #1 or “stem-loop” for transgene #2; green block arrows indicate activation of the reporter by endogenous *eve* enhancers) consistent with the results shown in Fig 1.

**Fig 3:**
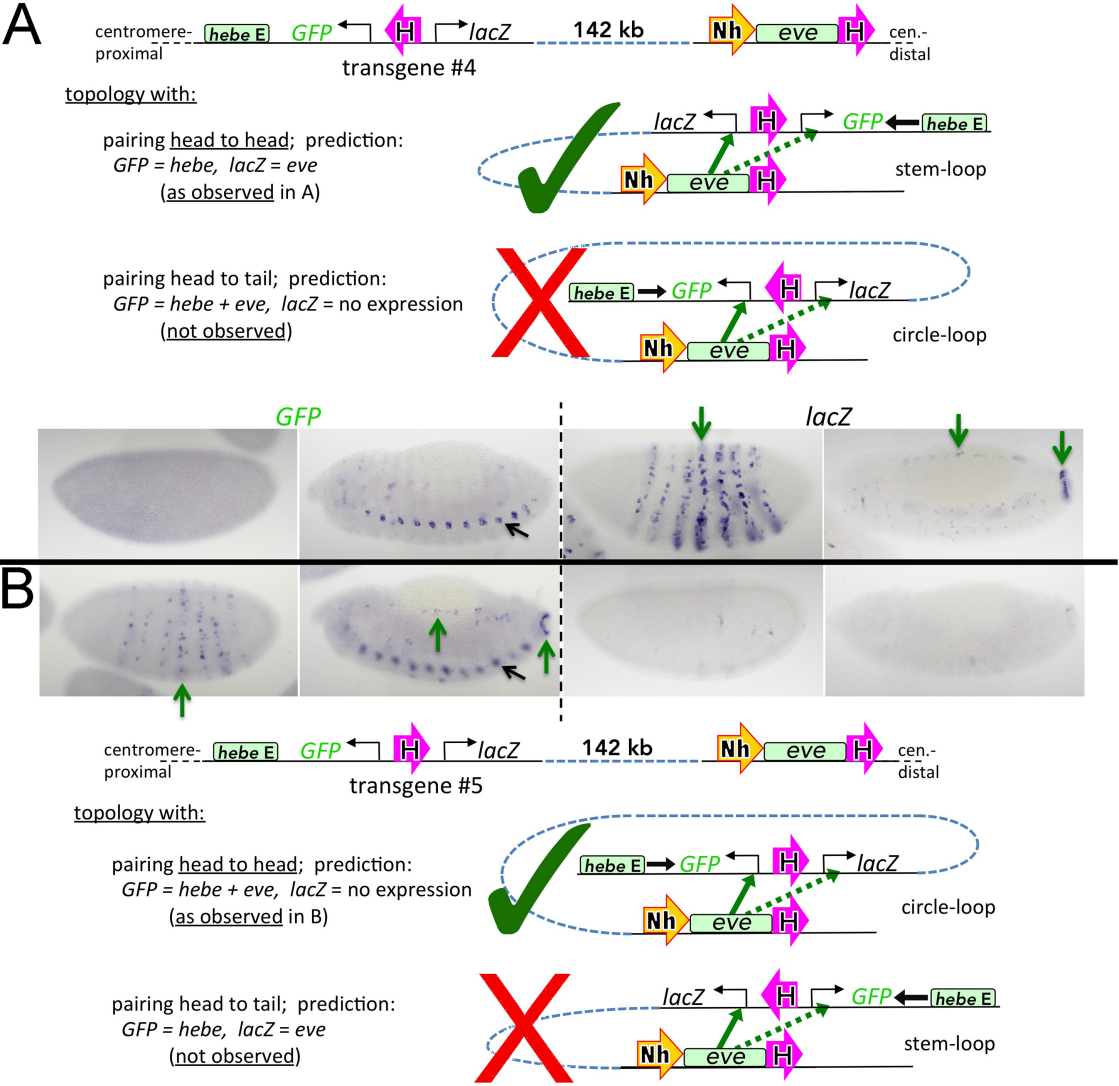
Orientation-specific facilitation of long-range communication by Homie. Dual reporter RNA expression 142 kb from *eve*. **A** and **B** have opposite orientations of Homie, resulting in distinctly different topologies that give rise to different reporter gene activation patterns. Large green check marks indicate which of the two possible topologies is consistent with the observed activation pattern. Within the diagrams, magenta block arrows represent Homie; smaller green and dotted green block arrows represent strong and weak activation by endogenous *eve* enhancers, respectively. Stages 5–6 (left panel in each quadrant) and 13 (right panel in each quadrant) are shown for each reporter. Within the micrographs, green arrows indicate aspects of *eve*-like expression; black arrows indicate elements of *hebe*-like expression (repeated in each segment).

The experiments in Fig 1 demonstrate that reporter activation by the enhancers in the *eve* locus is independent of the orientation of the Homie-*lacZ* transgene in the chromosome. However, this doesn’t mean that reporter activation is independent of the relative orientation *within the transgene* of Homie and the reporter. To explore this possibility, we generated a transgene with two divergently transcribed reporters, *lacZ* and *GFP* (both are driven by the same *eve* basal promoter, see Materials and Methods). We then inserted Homie in both orientations between the two reporters. Fig 2A shows that in the endogenous *eve* locus, Homie is located downstream of the *eve* transcription unit in what we have designated as the “forward” 5’→3’ orientation (→). In transgene #4 (Fig 2A), using this same 5’→3’ convention for the relative orientation of the Homie insulator, the *lacZ* reporter would be located 5’ with respect to Homie. Thus, in this transgene the relationship between the reporter and Homie is just like the endogenous *eve* locus where the *eve* gene is located 5’ to Homie. The *eve-GFP* reporter is in turn located 3’ to the Homie insulator in the same relative position as the *TER94* gene is with respect to the endogenous Homie. In transgene #5 (Fig 2A), the 5’→3’ orientation of Homie is flipped, so that *GFP* is now located 5’ relative to the Homie insulator, while *lacZ* is 3’. Each transgene was then inserted at -142 kb so that *GFP* is on the same side of Homie as the *hebe* enhancer, while *lacZ* is separated from the *hebe* enhancer by Homie (see diagrams in Fig 3A and 3B).

The two reporters in transgene #4 differ dramatically in their pattern(s) of expression (Fig 3A). In the case of the *lacZ* reporter, the *eve* enhancers activate expression in stripes in the early embryo, as well as in the CNS, mesoderm, and anal plate during mid-embryogenesis (green arrows). The *lacZ* reporter is not, however, activated by the *hebe* enhancer, as it is insulated by Homie. A quite different result is observed for the *GFP* reporter. First, unlike *lacZ*, it is not subject to regulation by the *eve* enhancers. Second, it is subject to regulation by the *hebe* enhancer (Fig 3A, black arrow). In transgene #5, the target for regulatory interactions with the *eve* locus is reversed (Fig 3B). Here, *GFP* is regulated by the *eve* enhancers (green arrows), while *lacZ* is not. And, since the orientation of the transgene in the chromosome remains the same, the *hebe* enhancer still activates *GFP* (Fig 3B, black arrow), while Homie blocks it from regulating *lacZ*.

These findings indicate that pairing interactions between the transgene Homie and the *eve* locus are orientation-specific. With respect to the endogenous Homie insulator (see below for Nhomie), the pairing interactions could be head-to-head or head-to-tail (Fig 3 diagrams). In the simplest topological model, head-to-head interactions predict that the *lacZ* reporter will be activated by *eve* enhancers when the 5’→3’ orientation of Homie in the transgene places this reporter 5’ of Homie, just as the *eve* enhancers in the *eve* locus are 5’ of the endogenous Homie (Fig 3A, transgene #4, “stem-loop” topology). The *GFP* reporter will be activated when the orientation of the transgene Homie is reversed (Fig 3B, transgene #5, “circle-loop” topology). The opposite pattern of activation is expected if Homie interactions are head-to-tail (Fig 3A and 3B, bottom diagrams). In each case, the topology of one of the variants is a stem-loop, while the topology of the other variant is a circle-loop. As can be seen from the expression patterns in Fig 3, it is head-to-head pairing between the transgene and endogenous Homie that fits the pattern of activation.

Why are the regulatory interactions in Fig 3 orientation-dependent, while those in Fig 1 are not? The difference lies in how we altered the orientation of the insulator in the two experiments. In the experiments in Fig 3, the 5’→3’ orientation of the Homie insulator in the transgene with respect to the two reporter genes was reversed. In contrast, in the experiments in Fig 1, the relative 5’→3’ orientation the Homie insulator with respect to the reporter was maintained (the reporter is 5’ with respect to Homie), while the orientation of the entire transgene was flipped. In the experiment in Fig 1 (illustrated in Fig 2B) head-to-head pairing between the Homie insulators in the transgene and the *eve* locus generates either a circle-loop (top, transgene #1) or a stem-loop (bottom, transgene #2). However, in both cases, the *eve* enhancers are brought into close proximity to the *lacZ* reporter. Note that as in Fig 3, the structure of the loops predicted for head-to-tail pairing of the Homie insulators in the Fig 1 experiments would place the *lacZ* reporter and the enhancers in the *eve* locus on opposite sides of the paired insulators, which would not be conducive for productive regulatory interactions (not illustrated).

### Nhomie mediates long-distance regulatory interactions with *eve* and insulates a reporter

**Fig 4:**
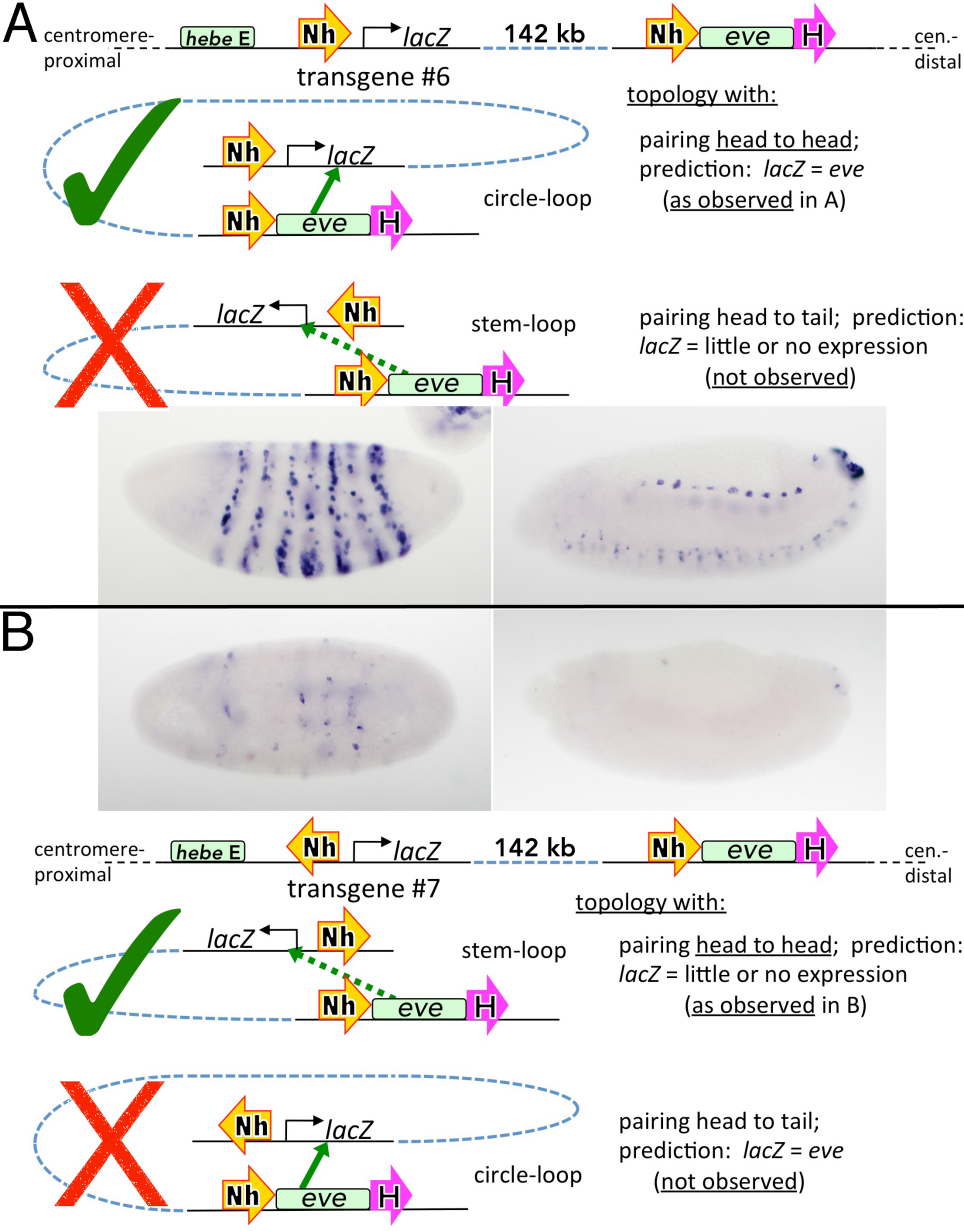
Nhomie facilitates long-range interactions of a reporter with *eve* in one orientation only. Reporter RNA in embryos (stages 5 and 12–13) is shown (transgenes at -142 kb, as in Fig 3) with opposite orientations of Nhomie (yellow block arrows) in **A** and **B**. Note that the expression seen in A is like that for transgene #2 of Fig 1A, and does not include expression driven by the *hebe* CNS enhancer, indicating that its activity is blocked by Nhomie (this blocking activity is also apparent in Fig 4B).

Our 3C experiments identified a second element, Nhomie, in the *eve* locus that interacts physically with Homie at -142 kb (Fig 1C). We wondered whether Nhomie could also promote long distance regulatory interactions and function as an insulator. To test for these activities, we combined the Nhomie insulator with the *lacZ* reporter (Fig 2A). The Nhomie:*lacZ* transgene was inserted at -142 kb so that Nhomie is located between the *lacZ* reporter and the *hebe* enhancer (Fig 4A diagram, transgene #6). Since the relative orientation of Homie and the reporter was critical for *productive regulatory* interactions, we tested Nhomie in both orientations relative to *lacZ*. Using the same convention as was used for Homie, the 5’→3’ orientation of Nhomie in the endogenous locus places the *eve* enhancers and the *eve* gene 3’ of Nhomie. In transgene #6 (Fig 4A), the 5’→3’ orientation of Nhomie places *lacZ* is the same position relative to Nhomie as are the *eve* enhancers and *eve* gene in the endogenous locus: the reporter is located 3’ relative to Nhomie. In transgene #7 (Fig 4B), Nhomie is in the reverse orientation with respect to *lacZ*. In this case, the 5’→3’ orientation of Nhomie places the reporter 5’ with respect to the insulator.

Our experiments show that Nhomie shares many properties with Homie. Like Homie, it functions as an insulator and blocks the *hebe* enhancer from activating the reporter (Fig 4). It is also able to mediate long-distance regulation of the reporter by *eve* enhancers (Fig 4A). Moreover, as for Homie, these regulatory interactions depend upon the orientation of Nhomie relative to *lacZ*. However, the orientation of Nhomie with respect to the reporter that engenders robust activation is the opposite that of Homie. For Homie, the reporter is activated when it is located 5’ with respect to the orientation of the insulator, just like the *eve* gene is 5’ of the endogenous Homie. By contrast, for Nhomie, the reporter is activated when the orientation of the insulator places it 3’ relative to Nhomie; again, just as the *eve* gene is located 3’ relative to the endogenous Nhomie.

### Homie mediates homolog alignment and pairing

Our 3C experiments show that Homie at -142 kb physically interacts with Homie at the 3’ end of the *eve* locus (Fig 1C). It is clear from bypass experiments that self-interactions like that observed for Homie are not unusual, but instead are a characteristic property of fly insulators [35–37,41,42]. However, these transgene assays artificially juxtapose homologous partners in *cis*, as we have done here. In the endogenous setting, homologous partners are only present on the other homolog, and it is in this context that homologous interactions would be biologically relevant. Given that most fly insulators self-interact head-to-head, a plausible idea is that insulators are the elements responsible both for locally aligning homologs in precise register and for maintaining their stable association.

The classical evidence for homolog pairing in Drosophila is transvection [43–45]. Transvection is a regulatory interaction that occurs in *trans* rather than in *cis*, and requires local pairing of homologs. Typically two mutant alleles complement because the regulatory elements on one homolog activate the gene on the other homolog. Complementation is lost when pairing of the two alleles is disrupted by chromosomal rearrangements [46]. While a special combination of mutations is generally required to detect transvection, *trans-*regulatory interactions are clearly important for achieving appropriate levels of gene activity in wild-type flies [47].

The hypothesis that homologous insulator:insulator interactions are responsible for the pairing of homologs in register makes two predictions. First, placing homologous insulators in *trans* should promote transvection. Second, if the homologous interactions of the test insulator are orientation-dependent, transvection is expected to be greater when both copies are oriented in the same direction than when they are oriented in opposite directions. This is expected because self-pairing interactions are likely to be head-to-head rather than head-to-tail. There are two reasons behind this expectation. One is that the self-interactions detected in insulator bypass experiments are typically head-to-head, not head-to-tail [41,48,49]. The other is that head-to-tail self-interactions between endogenous insulators on each homolog would likely interfere with homolog alignment as well as transvection.

**Fig 5:**
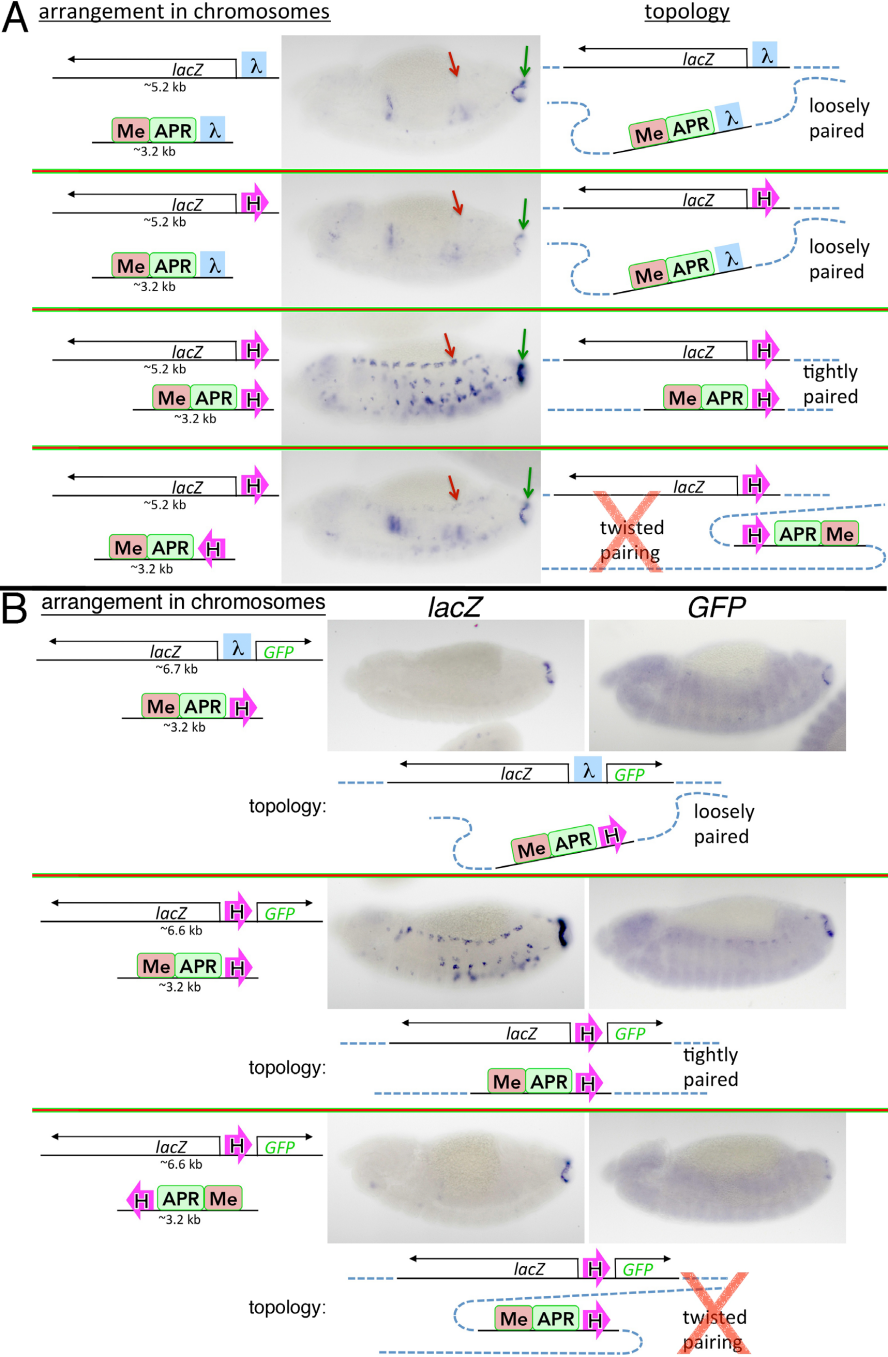
Homie-Homie pairing facilitates transvection. Transgene reporters and enhancers (*eve* mesodermal, “Me”, and anal plate ring, “APR”) are in *trans* on homologous chromosomes at the 23C4 attP site. Block arrows show Homie orientation. Stage 13 embryos are shown. A red “X” in a topology diagram indicates that this topology, predicted based on head-to-head insulator pairing, may not form because it tends to disrupt homolog pairing. **A:** single reporter RNA in the APR (green arrow), and the mesoderm (red arrow). **B:** dual reporter RNA (*lacZ* or *GFP*, as indicated at the top) from the diagrammed transgene combinations.

To test these predictions, we generated two transgenes, one containing the *eve* APR and mesoderm (Me) enhancers [25], and the second containing the *lacZ* reporter. The transgenes were inserted into a site far away from endogenous *eve* (on a different chromosome arm, at cytological location 23C4, where we do not see interactions with endogenous enhancers [26]), oriented so that both the enhancers and reporter are on the centromere-distal side of their respective transgene (Fig 5A). In the first experiment, the enhancer transgene had λ DNA, while the reporter had either DNA or Homie. Since there are insulator-like elements near the 23C4 attP site (one ~50 bp distal to the attP site, another ~8 kb proximal) [40], we expected to see some transvection [50,51] when either:*lacZ* or Homie:*lacZ* is *trans* to the:enhancer transgene. Fig 5A (top two panels) shows that the APR enhancer weakly activates *lacZ* (green arrows), while there is virtually no Me-driven expression (red arrows). As predicted, the presence of a forward-oriented Homie in the enhancer transgene substantially augments transvection (Fig 5A, 3^rd^ panel). Not only is APR expression much stronger (green arrow), but Me-driven expression is also clearly observed (red arrow).

To confirm that stable pairing is head-to-head, we reversed Homie in the enhancer transgene (maintaining the overall transgene orientation in the chromosome). In this configuration, head-to-head pairing would introduce an S-shaped double loop. As illustrated in Fig 5A (“twisted pairing”), this would place the reporter on the opposite side of the paired insulators from the transgenic enhancers. This configuration would not be expected to increase enhancer-reporter interactions. Consistent with this prediction, reporter expression is about the same (Fig 5A, bottom panel) as in the negative controls carrying DNA (Fig 5A, top two panels). Alternatively, the need to form such a double loop might make this pairing interaction less stable than for the other orientation, when the head-to-head pairing reinforces the normal pairing of the homologs (“tightly paired” in the diagram). In fact, evidence below is more consistent with such “twisted pairing” interactions forming only transiently, or not at all (hence the red “X” in the diagram for “twisted pairing”). This is in line with the expectation, stated above, that head-to-head self-interactions between endogenous insulators mediate homolog alignment and pairing, while head-to-tail self-interactions are incompatible with smooth alignment and tight pairing.

To further explore the relationship between pairing direction and transvection, we generated dual reporters with divergently transcribed *GFP* and *lacZ* that have either DNA or Homie inserted between the reporters (Fig 5B). When the DNA:dual reporter is *trans* to the Homie-enhancer transgene, the APR enhancer weakly stimulates *lacZ and GFP* in the APR, while neither reporter is activated by the Me enhancer. The addition of Homie to the reporter (in the same orientation in the chromosome as that of Homie in the enhancer transgene) substantially enhances APR *lacZ* transcription, and turns on *lacZ* in the mesoderm. By contrast, there is only a slight increase in APR *GFP* expression, while mesoderm expression is detectable, but only weakly. The differences in transvection for the two reporters are consistent with the topology generated by head-to-head, not head-to-tail pairing (Fig 5B, “tightly paired”).

We also combined the dual reporter with an enhancer transgene in which the entire transgene containing Homie and the enhancers are flipped (Fig 5B, bottom panel). Head-to-head pairing of Homie would generate an S-shaped double loop (as diagrammed in Fig 5B, “twisted pairing”). In this case, there is little or no enhancement of transvection for either reporter, suggesting that the introduction of such a double loop between the paired homologs either is unstable or does not form (indicated by the red “X” in the diagram for “twisted pairing”).

We note that there are some subtle differences in the expression patterns for transgene combinations in which transvection is not significantly enhanced. This includes all the cases where our topology diagrams are labeled as “loosely paired” or “twisted pairing”. These differences may be due to a combination of several factors, such as differences in the size of the transgenes, weak or unstable interactions with insulators near the site of transgene insertion, or the shielding of transgenic reporters from position effects that weakly upregulate or downregulate reporter activity.

### Nhomie promotes homolog pairing

**Fig 6:**
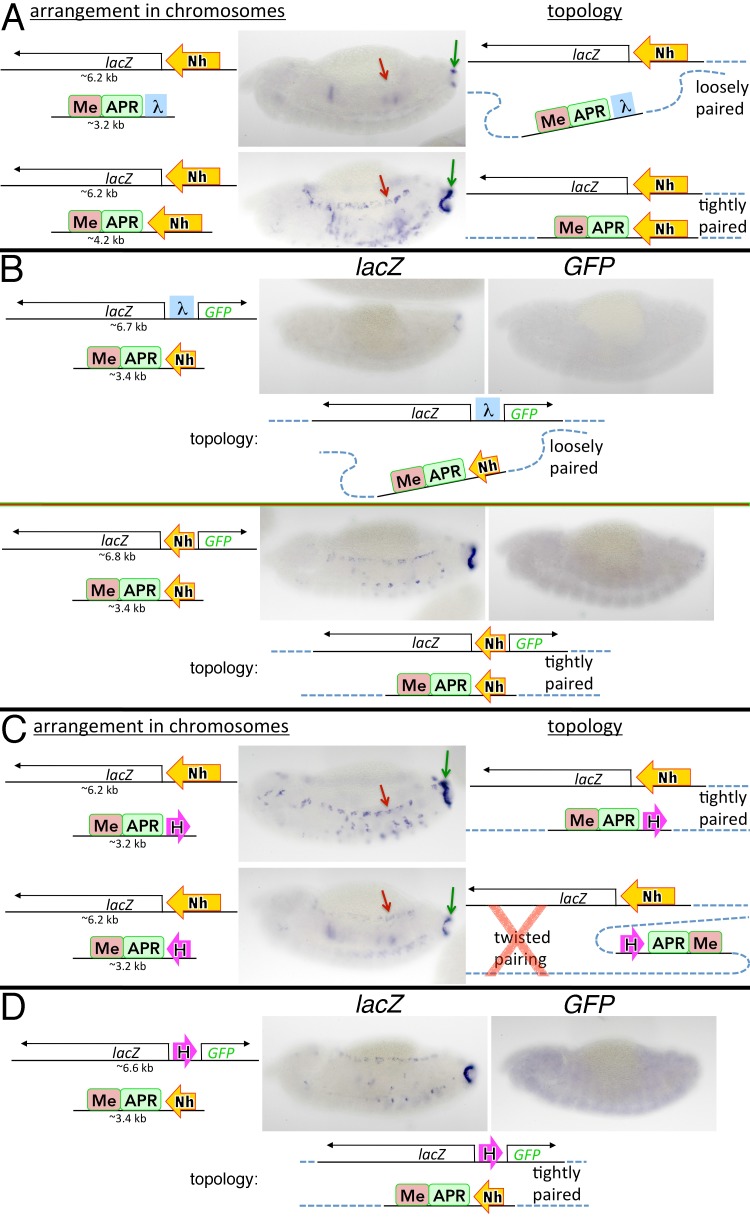
Nhomie-Nhomie and Nhomie-Homie pairing facilitate transvection. Transgenes are as in Fig 5, except that block arrows labeled “Nh” are Nhomie. Stage 13 embryos are shown. **A and C:** single reporter RNA in the APR (green arrow) and the mesoderm (red arrow). A red “X” in the “twisted topology” diagram indicates that this topology, predicted based on head-to-tail pairing between Homie and Nhomie, may not form in this case because it tends to disrupt homolog pairing. The chromosomal fragment used here for Nhomie is the same as that used in Fig 4 (1329 bp, see S1 and S2 Figs). **B and D:** dual reporter RNA (*lacZ* or *GFP*, as indicated at the top) from the diagrammed transgene combinations. The chromosomal fragment used here for Nhomie is a shorter version (603 bp) that retains most of the long-range interaction activity of the larger fragment (in the same assay as in Fig 4, shown in S2 Fig).

We next tested whether Nhomie self-interactions in *trans* also induce transvection. Nhomie was oriented in the single reporter transgene so that the *lacZ* reporter (diagrammed in Fig 6A) is 3’ with respect to Nhomie. It was then combined in *trans* with an enhancer transgene that had or Nhomie (in the same 5’→3’ orientation in the chromosome) so that the two enhancers are 3’ of Nhomie (Fig 6A). In the:Nhomie combination, the APR enhancer drives only weak expression, and activation by the Me enhancer is not seen. As would be predicted if head-to-head pairing aligns the enhancers and the reporter, *lacZ* expression is substantially elevated in the Nhomie:Nhomie combination. This conclusion is confirmed by the dual reporter assay. As shown in the lower half of Fig 6B, head-to-head pairing of Nhomie in the enhancer and dual reporter transgenes would juxtapose the Me and APR enhancers with the *lacZ* reporter, while the *GFP* reporter would be separated from the enhancers by the paired Nhomie insulators. In this configuration, the Me and APR should preferentially drive *lacZ* expression, not *GFP* expression, and this is what is observed.

### Transvection induced by combining Homie and Nhomie

While Homie-Homie or Nhomie-Nhomie self-interactions normally occur at the endogenous *eve* locus only in *trans*, this is not the case for Nhomie-Homie interactions. Heterologous interactions between neighboring insulators in *cis* are thought to be responsible for subdividing chromosomes into a series of topologically independent domains, and are expected to occur all along the chromosome. Like self-interactions, heterologous interactions are known to be specific [37,42,52,53], and consequently are likely also orientation-dependent. For heterologous insulators interacting in *cis*, we define their endogenous directionalities to be the same. That is, the arrows that represent them point in the same “forward” direction along the chromosome (as in Figs 1–4 for endogenous Nhomie and Homie). Using this convention, at the endogenous *eve* locus, head-to-tail interactions between Nhomie and Homie would generate a stem-loop, while head-to-head interactions would generate a looped circle or “circle-loop.” To test whether these two insulators can interact with each other independently of the *eve* locus, and (if so) determine their orientation dependence, we combined a Nhomie-*lacZ* reporter with two different Homie-enhancer transgenes. In the one in which the enhancers are 5’ of Homie (Fig 6C, top panel), head-to-tail pairing with *Nhomie* should align the enhancer and reporter, and favor transvection. When the enhancer transgene has Homie in the reverse orientation (Fig 6C, bottom panel), enhancer-reporter alignment would be favored by head-to-head pairing. Fig 6C shows that Nhomie and Homie can pair with each other in a foreign context (top panel), and that transvection is favored by head-to-tail pairing (top panel vs. bottom panel). These findings parallel those for self-pairing (Figs 5 and 6A and 6B), *except that* heterologous pairing is head-to-tail rather than head-to-head.

To confirm these results, we combined the dual *lacZ*, *GFP* reporter containing Homie with an enhancer transgene containing Nhomie. As illustrated in Fig 6D, head-to-tail pairing of Nhomie and Homie would juxtapose the enhancers with *lacZ*, while head-to-head pairing would juxtapose the enhancers with *GFP*. Consistent with head-to-tail pairing, *lacZ* transvection is stimulated, while *GFP* is not (compare 6D with the control in the upper half of 6B).

### Homie and Nhomie long-distance interactions are *eve*-independent

**Fig 7:**
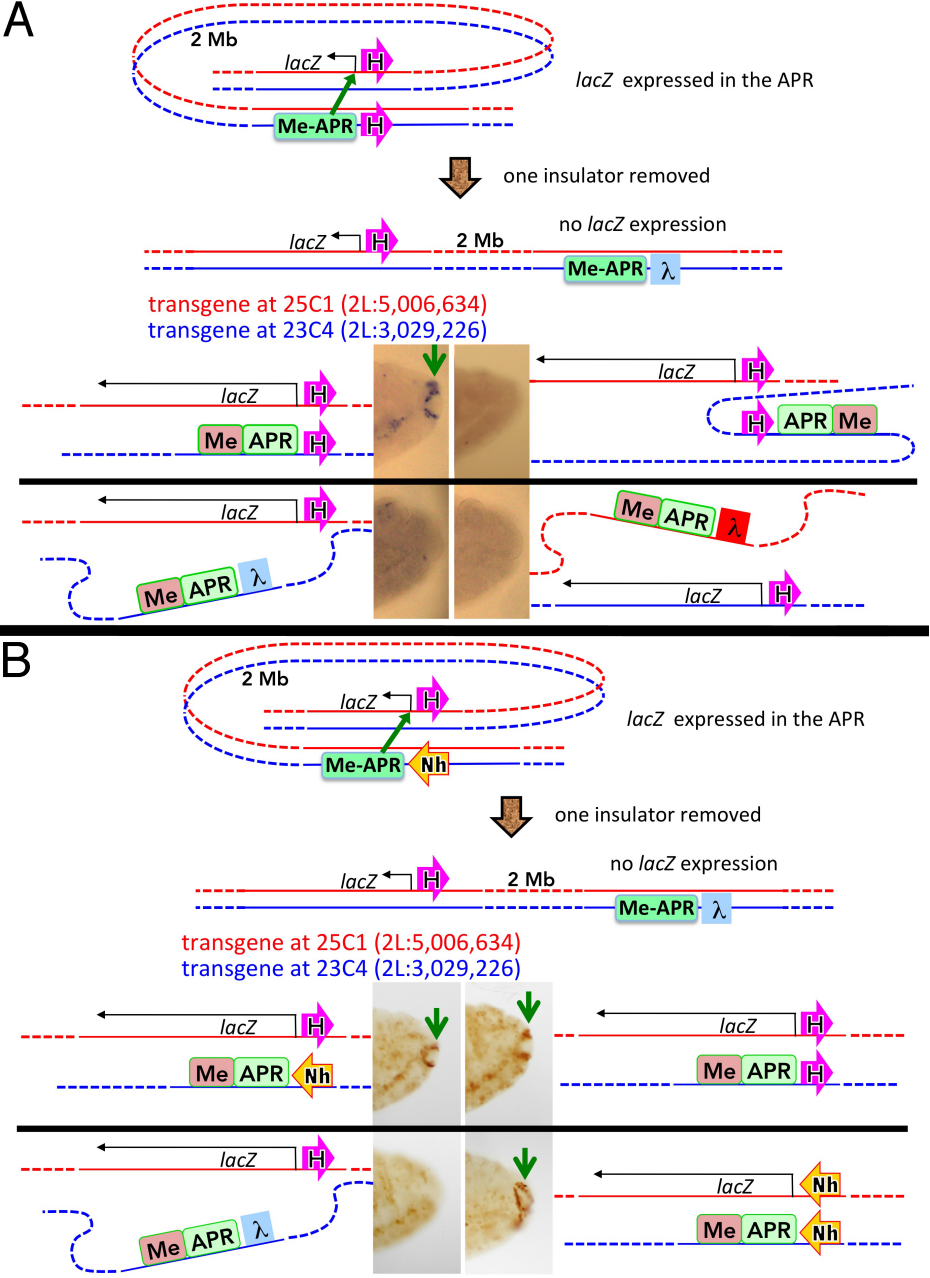
Specific insulator interactions bring distant regions together in *trans*. Each construct diagrammed in red is at 25C1, and in blue at 23C4. **A:** Homie-Homie pairing. Top, overview: reporter (*lacZ*) with Homie (magenta arrow), and enhancer transgene (“Me-APR”) with either Homie or DNA (as diagrammed) at sites ~2 Mb apart. In doubly transgenic embryos, *lacZ* RNA is seen in the APR (green arrow, upper left panel) only when both transgenes carry Homie. However, when Homie is inverted within the enhancer transgene (upper right panel), expression is not seen, confirming the head-to-head orientation specificity of Homie-Homie pairing. **B:** Homie-Nhomie and Nhomie-Nhomie pairings. Top: overview, as in A; “Nh” is Nhomie. Bottom: β-gal (*lacZ* protein) staining in the APR (green arrows) is seen, facilitated by either head-to-head Homie or Nhomie self-pairing, or by head-to-tail Nhomie-Homie pairing, as diagrammed.

The insulator interactions in the transvection assay are local and likely facilitated by homolog pairing. To confirm that the *eve* insulators can interact *specifically* with themselves and with each other over large chromosomal distances, we took advantage of attP 25C1, located 2 Mb distal to 23C4. A Homie:*lacZ* transgene was inserted at 25C1. It was combined with an enhancer transgene at 23C4 containing either DNA or Homie (Fig 7A). No interaction between the transgenes is evident with the DNA control or when is replaced by the *su(Hw)* insulator. On the other hand, when both the reporter and the enhancer have a Homie insulator, the APR enhancer is able to activate *lacZ* expression (Fig 7A, upper left panel). This result is consistent with previous studies which showed that APR was the only enhancer in the endogenous *eve* locus that could act over distances >1 Mb with Homie-carrying transgenes [26]. As would be expected from the orientation dependence of insulator self-pairing, when Homie is inverted within the enhancer transgene (Fig 7A, upper right panel), expression is not seen, confirming that Homie-Homie pairing is head-to-head.

We also tested whether Nhomie can mediate distant regulatory interactions either with itself or with Homie. In the two transgenes used to test Nhomie self-interactions, the enhancers or reporter, respectively, are each located 3’ relative to the adjacent insulator. These Nhomie transgenes were inserted (separately) at 23C4 and 25C1, then crossed into the same animals. Fig 7B shows that Nhomie:Nhomie interactions can mediate long-distance activation of *lacZ* by the APR enhancer (Fig 7B, lower right). Nhomie also pairs with Homie, enabling the APR enhancer in the Nhomie transgene at 23C4 to activate a Homie-*lacZ* reporter at 25C1 (Fig 7B, upper left). As illustrated in Fig 7, these interactions are all consistent with the orientation dependence seen in the other assays, namely head-to-head self-pairing and head-to-tail heterologous pairing.

### Nhomie and Homie physically interact in the endogenous *eve* locus

The experiments described above indicate that Nhomie and Homie must be able to physically pair with each other, and do so in a head-to-tail orientation. In the endogenous locus, head-to-tail pairing would generate a stem-loop containing the *eve* transcription unit and its associated enhancers and Polycomb silencers, linked together at the base by the Nhomie and Homie insulators. 3C experiments with Homie as the anchor confirm that Nhomie and Homie contact each other in the *eve* locus (Fig 1D).

## Discussion

The importance of insulators in organizing eukaryotic chromosomes has been recognized since their discovery in the 1980’s. However, the principles underlying their architectural and genetic functions have not been fully elucidated. With this goal in mind, we asked how these elements shape two critical architectural features of chromosomes. The first is homolog pairing. Homologs pair in flies from the blastoderm stage onward, and the consequent *trans*-interactions are important for proper gene regulation. The phenomenon of homolog pairing is not unique to Drosophila [24,54]. Homologs are paired in lampbrush chromosomes of invertebrate and vertebrate oocytes. The second is the looped domain organization [20,21,55]. Although there is now compelling evidence that insulators subdivide chromosomes into topologically independent looped domains (and that these domains play a central role in gene regulation), the topology of the loops is unknown. Moreover, while the loops must emanate from the main axis of the chromosome, the relationships between the loops, the insulators that delimit them, and the main chromosomal axis are not understood. As homolog pairing is more straightforward and the likely mechanism better documented, it is considered first.

### Insulators and homolog pairing

Homolog pairing requires mechanisms for aligning homologs in precise register, and maintaining their stable association. While many schemes are imaginable, the simplest utilizes elements distributed along each homolog that have self-interaction specificity. Such a mechanism would be consistent with the persistence of local pairing and transvection in chromosomal rearrangements [44,56–60]. It would also fit with studies on the pairing process [56,61,62]. Self-association of pairing elements would locally align sequences in register, and ultimately link homologs together along their entire length. In this mechanism, self-association must be specific *and also* directional, namely head-to-head. This avoids the introduction of unresolvable loops and maximizes pairing for transvection.

In Drosophila, the homing of P-element transgenes, in which normally random insertion becomes targeted, suggested the ability of genomic elements to self-interact. Such a homing activity was found in the *engrailed* locus for a region that includes two PREs [63–65], and later studies showed that some insulators [26,66,67] and a promoter region [68] also possess homing activity. The self-interaction implied by homing suggests that these elements may facilitate homolog pairing. However, in contrast to PREs and promoters, insulators have consistently been found to engage in specific self-interactions (see below). Thus, among the known elements in the fly genome, insulators are the best candidates to align homologs in register and maintain pairing [20,21]. Moreover, genome-wide chromatin immunoprecipitation experiments (ChIPs) show that insulators are distributed at appropriate intervals along each chromosome [18,19].

A role in homolog pairing was first suggested by the discovery that the *su(Hw)* and *Mcp* insulators each can mediate regulatory interactions between transgenes inserted at distant sites [69,70]. The *Fab-7* insulator can also mediate long-range regulatory effects [71]. Further evidence that self-association is characteristic of fly insulators came from insulator bypass experiments [35,36]. These experiments showed that bypass is observed when an insulator is paired with itself, while heterologous combinations are less effective or don’t give bypass [37,41,42,48,72,73]. Moreover, self-pairing is, with few exceptions, head-to-head.

That insulators mediate homolog pairing through specific self-interactions is further supported by our studies. Using a classical transvection assay, we found that Homie-Homie and Nhomie-Nhomie combinations stimulate *trans*-regulatory interactions between enhancers on one homolog and a reporter on the other (Figs 5, 6A and 6B). Moreover, the parameters that favor transvection dovetail with those expected for a pairing mechanism based on insulator self-interactions in *trans*. First, the two insulators must be in the same orientation. When they are in opposite orientations, transvection is not enhanced (or enhancement is much weaker, Fig 5). Second, the enhancers and reporter must be located on the same side (centromere proximal or distal) of the insulators (Figs 5, 6A and 6B). In addition to transvection, Homie and Nhomie also engage in highly specific and directional distant regulatory interactions (Fig 7).

While there is compelling evidence that insulator self-interactions are responsible for homolog pairing, many issues remained unresolved. Perhaps the most important is the nature of the code used for self-recognition and orientation. The best hint comes from bypass experiments using multimerized binding sites for Su(Hw), dCTCF, or Zw5. Homologous multimer combinations give bypass, while heterologous combinations do not. However, bypass is observed for composite multimers when they are inserted in opposite orientations (e.g., Su(Hw) dCTCF ↔ dCTCF Su(Hw)), but not the same orientation (e.g., Su(Hw) dCTCF **→→** Su(Hw) dCTCF) [53]. These findings argue that the identity and order of proteins bound to the insulator determine its self-association properties.

### Topology of looped domains and the higher order architecture of the chromosome

The first direct evidence that insulators generate loops came from 3C experiments on the mouse β-globin and the fly 87A7 heat shock loci [23,74]. These studies suggested that physical interactions between adjacent insulators in *cis* could subdivide chromosomes into looped domains. Subsequent work has confirmed this conclusion [17]. However, while these experiments demonstrate that *cis* insulator interactions generate loops, they provided no information about the topology of these loops, or how they are arranged.

**Fig 8:**
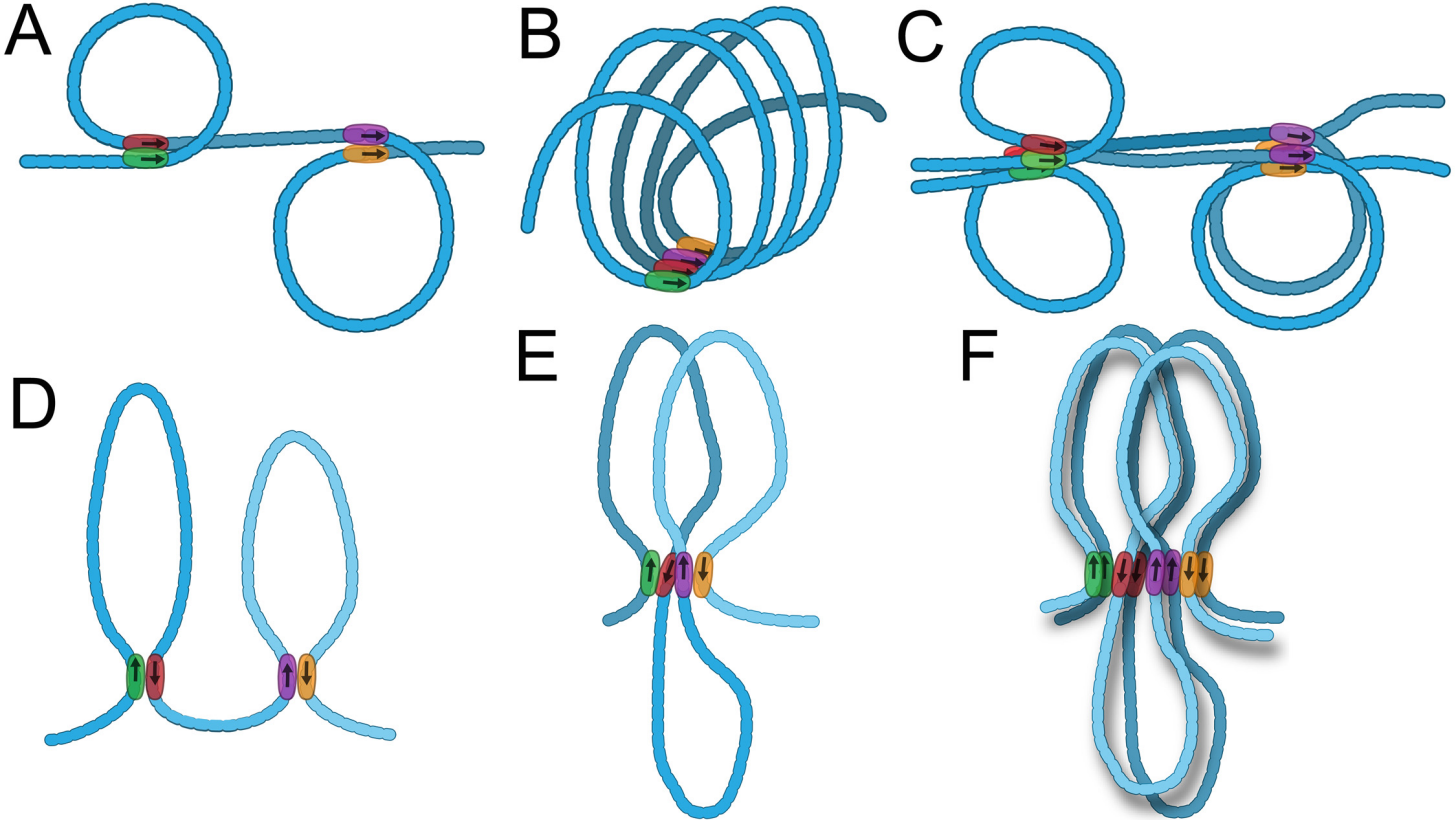
Chromosome architecture: pairing head-to-head and head-to-tail in *cis*. **A-C: Head-to-head insulator pairing in *cis*.** A: head-to-head pairing of two sets of insulators (green with red, purple with gold). Circle-loop on left is left-handed, circle-loop on right is right-handed. B. Head-to-head insulator pairing defines the main chromosomal axis. Circle-loops are all right-handed in this illustration. C: homolog pairing of chromosome in A, with head-to-head insulator interactions both in *cis* and in *trans*. Left: top circle-loop is left-handed, bottom circle-loop is right handed. Right: both circle-loops are right-handed. **D-F: Head-to-tail insulator pairing in *cis*.** D: head-to-tail pairing of two sets of insulators. E: head-to-tail insulator pairing also defines the main chromosomal axis. F: homolog pairing of chromosome in E, with head-to-tail insulator pairing in *cis* and head-to-head insulator pairing in *trans*.

*Cis* interactions could, a priori, be either head-to-head like self-association in *trans*, or head-to-tail. The consequences are quite different. Head-to-head interactions generate a circle-loop, while head-to-tail interactions generate a stem-loop (Fig 8A and 8D, respectively). If heterologous insulators interact with only one specific partner, the circle-loop or the stem-loop will be linked to neighboring circles or stem-loops by loops without anchors. These unanchored loops would correspond to the main axis of the chromosome, and the circle-loops or stem-loops would then protrude from the main axis in a random orientation and at distances determined by the length and compaction of the unanchored loops.

On the other hand, if insulators in a chromosomal segment are able to interact with both of their neighbors, then the main axis of the chromosome in this region would be defined by the insulators. Quite different structures are predicted for head-to-head and head-to-tail interactions (Fig 8B and 8E). Head-to-head would give a series of variably sized circle-loops linked together at their base by an array of interacting insulators. The base would correspond to the main axis of the chromosome, and each circle-loop would extend from one side of the main axis to the other. If the direction of coiling were always the same, this would give a structure resembling a helix anchored to a rod (Fig 8B). If the direction of coiling were random, the structure would be more complicated and variable, since neighboring circle-loops could extend out from the main axis in either the same or the opposite direction (not illustrated). The loop-axis relationship would be more regular for head-to-tail insulator pairing in *cis*. Adjacent stem-loops would extend out from the main axis in opposite directions much like the lampbrush chromosomes formed when haploid sperm heads are injected into amphibian oocytes (Fig 8E) [75]. This stem-loop organization would also fit with the radial loop model proposed by Laemmli and others for the first level of folding of metaphase chromosomes [7,11].

Since our experiments show that Homie-Nhomie association is head-to-tail, the topology of the *eve* locus *in vivo* is a stem-loop, not a circle-loop. This finding raises a number of questions. Perhaps the most important is whether head-to-tail interactions are the rule rather than the exception. While the orientation dependence of homologous interactions has been extensively investigated, there have been no systematic studies on interactions between neighboring insulators. However, there are reasons to think that *cis* interactions are more likely head-to-tail than head-to-head. One is homolog pairing. As mentioned above, the circle-loops formed by head-to-head interactions can coil in either direction, either left-handed or right-handed. If coiling were random, then about half of the circle-loops on each homolog would be coiled in opposite directions. In this case, head-to-head pairing of homologous insulators in each homolog would generate a structure in which the circle-loops would point in opposite directions (Fig 8C, left circles). This topology would not be compatible with transvection. Coiling of the circle-loops in the same direction on both homologs would permit interdigitation of one circle-loop inside the other (Fig 8C, right circles); however, the chromatin fiber from the inside circle-loop would need to cross in on one side and out on the other. If the main axis of the chromosome in the paired region is defined by a series of interacting insulators in *cis*, then generating a topology permissive for transvection (not illustrated) would require coiling of successive homologous circle-loops on each homolog in the same direction, one inside the other (Fig 8C, right circles).

These topological issues aren’t encountered when heterologous insulator interactions in *cis* are head-to-tail. Head-to-head pairing of homologous insulators in *trans* would bring regulatory elements and genes in the two homologous stem-loops into close proximity. Alignment of the two homologs is straightforward whether or not the main axis of the chromosome is defined by a series of interacting insulators (Fig 8F illustrates one of these cases). Alternating loops extending upwards and downwards from the main axis of the chromosome would be directly aligned when homologous insulators pair head-to-head in *trans*.

While the requirements for aligning and pairing homologs would appear to favor stem-loops between heterologous insulators in *cis* in flies, homolog pairing does not occur in vertebrates except in specialized cell types [76]. This could mean that circle-loops formed by *cis* interactions between heterologous insulators are permissible in vertebrate chromosomes. However, even in organisms in which homolog pairing doesn’t occur in somatic cells, it seems possible that *cis*-pairing interactions more commonly generate stem-loops than circle-loops. First, following DNA replication and before mitosis (during the S and G2 phases of the cell cycle), sister chromatids are aligned. Maintaining this alignment may facilitate epigenetic mechanisms that template chromatin structures from one cellular generation to the next, such as the copying of histone modifications onto both daughter chromosomes. The simpler topology of stem-loops could facilitate this sister chromatid pairing, as well as their separation during mitosis. Second, recent studies on the relationship between loop domains and CTCF insulators showed that in more than 90% of the cases, the CTCF binding sites on opposite ends of a loop are in opposite orientation [17]. Thus, assuming that the orientation of pairing is such that the CTCF sites are aligned in parallel to form the loop, pairing between CTCF insulators at the ends of the loop would generate stem-loops rather than circle-loops. If insulators form the main axis of the chromosome, there is an additional explanation for such a bias. As shown in Fig 8B, head-to-head pairing in *cis* could generate a series of circular loops that extend out from the same side of the main axis. This configuration would be favorable for crosstalk between regulatory elements and genes in adjacent loops. By contrast, head-to-tail pairing, where adjacent stem-loops extend out in opposite directions (Fig 8E), would disfavor crosstalk, helping to explain how insulators block enhancer-promoter communication between adjacent loops.

## Materials and Methods

### Transgenic flies

See S1 Fig for the Homie and Nhomie regions used, control DNA, and tag sequences. Reporters contain the *eve* basal promoter, -275 to +106bp from *eve* +1 (TSS), either the *lacZ* or *GFP* coding region, and the *eve* 3'-UTR, +1300 to +1525 bp. Enhancers are: *eve* APR,+3.0 to +4.1 kb; *eve* Me, +5.7 to +6.6 kb, each cloned in plasmid attB∆2 [32] for transgenesis [77] using ΦC31 [78]. Target sites were: -142 kb from *eve* [26]; 23C4 (2L;3029226), generated by us; and 25C1 [77]. Sequence coordinates are Flybase version dm6 [79]. Two genomic fragments used in this study that span the insulator protein binding region we call Nhomie, based on genome-wide studies [40], were found to have indistinguishable function in our assays (S2 Fig). The corresponding sequences are given in S1 Fig.

### Embryo analysis

RNA *in situ* hybridization and anti-β-galactosidase staining were as described [25]. In all cases, conclusions drawn were based on comparisons between control and experimental collections of embryos that were stained in parallel.

### High-resolution chromosome conformation capture

H3C analysis was performed as described [34], with the following modifications. Embryos (200 μl aged 0-6h at ~23°C) were cross-linked in either 2% or 3% formaldehyde for either 15 or 30 min (each gave similar results, and were included in the data presented), digested with 100U each of EcoRI (Roche) and MfeI (NEB) at 37°C overnight. About half of the material was ligated (Takara, 3500U) for >4 hr. at ~23°C, and incubated at 65°C overnight to reverse cross-links. Following RNase A (Roche, 40μg/sample) and proteinase K (Roche, 220μg/sample) digestions, purified DNA (20ng/reaction) was subjected to real-time PCR analysis using SYBR Green Master Mix (Roche).

All transgenes inserted at -142 kb used for 3C analysis had the same tag sequence, which was used as the anchor primer (Fig 1C), in combination with each of a series of accompanying primers from within the *eve* locus. To identify Homie-interacting regions within endogenous *eve*, an endogenous Homie fragment-specific primer was used as anchor (Fig 1D), along with the same series of accompanying primers. These sequences are given in S1 Fig.

PCR quantification was done as described [34], with the following set-up. The fragments in the *eve* locus created by EcoRI and MfeI digestion were cloned into anchor fragment-carrying plasmids, and served as standards for the expected ligation products. These plasmids were linearized and mixed with equimolar amounts of digested genomic DNA. Details of the various controls, such as the choice of primers and enzymes, were appropriate for each specific experiment [34]. Additional details are given in the legend to Fig 1.

## Supporting Information

S1 Fig Nucleotide sequences of primers, tags, insulators, and control DNA. Primers used for H3C, and sequences of transgenic insulators and control DNA are shown.
(XLSX)

S2 Fig Most of the long-range interaction activity of the original Nhomie genomic fragment resides in its *eve*-proximal half. The assay of Fig 4A (same transgenic reporter, same transgene insertion site, same transgene orientation in the chromosome, same orientation of the tested genomic fragment in the transgene as transgene #6, Fig 4A) was used to assess the two “halves” of Nhomie (1329 bp) for the ability to induce interactions between endogenous *eve* enhancers and a transgenic promoter-reporter located at -142 kb relative to the endogenous *eve* transcription start site. Embryonic stages are indicated on the left. The sequences tested (“1329 bp” indicates the original Nhomie fragment used in Fig 4, as well as in Fig 6A and 6C, “left half” indicates the *eve*-distal 729 bp, “right half” indicates the *eve*-proximal 603 bp used in Fig 6B and 6D) are given in S1 Fig. Note that only the “right half” induces expression in an *eve* pattern, while the “left half” not only has lost the ability to induce the long-range interaction with endogenous *eve*, but has also lost the ability to block interactions between the nearby *hebe* enhancer and the transgenic reporter (black arrow, as also seen in Fig 1A, transgenes #1 and #3, and in Fig 3B, transgene #5’s *GFP* reporter).
(TIF)
