# Supplementary figures and images for: Determinants of Chromosome Architecture: Insulator Pairing in cis and in trans
Source: PLoS Genet. 2016 Feb 24;12(2):e1005889. doi: 10.1371/journal.pgen.1005889 (PMC4765946; doi:10.1371/journal.pgen.1005889)

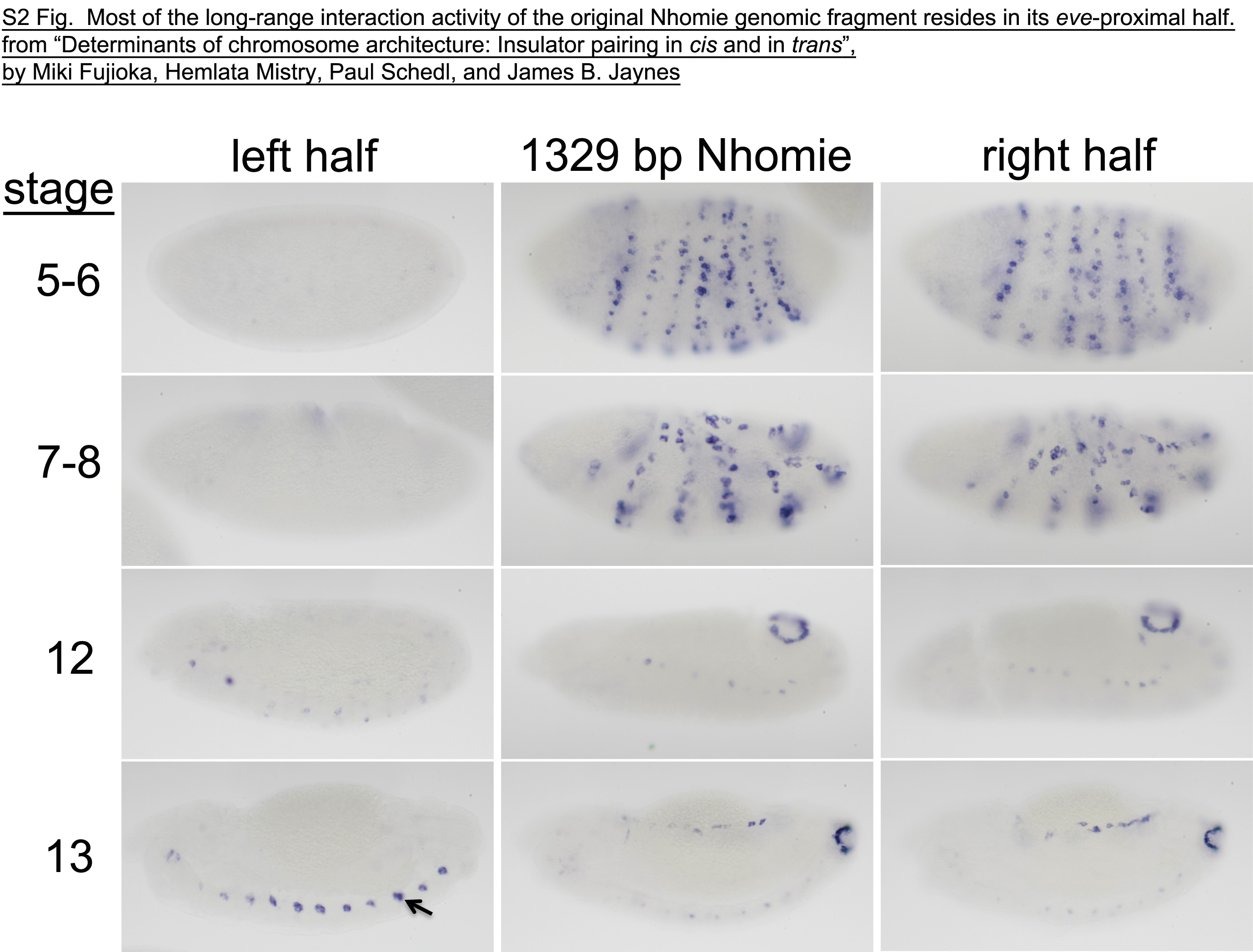

Supplement: S2 Fig — The assay of Fig 4A (same transgenic reporter, same transgene insertion site, same transgene orientation in the chromosome, same orientation of the tested genomic fragment in the transgene as transgene #6, Fig 4A) was used to assess the two “halves” of Nhomie (1329 bp) for the ability to induce interactions between endogenous eve enhancers and a transgenic promoter-reporter located at -142 kb relative to the endogenous eve transcription start site. Embryonic stages are indicated on the left. The sequences tested (“1329 bp” indicates the original Nhomie fragment used in Fig 4, as well as in Fig 6A and 6C, “left half” indicates the eve-distal 729 bp, “right half” indicates the eve-proximal 603 bp used in Fig 6B and 6D) are given in S1 Fig. Note that only the “right half” induces expression in an eve pattern, while the “left half” not only has lost the ability to induce the long-range interaction with endogenous eve, but has also lost the ability to block interactions between the nearby hebe enhancer and the transgenic reporter (black arrow, as also seen in Fig 1A, transgenes #1 and #3, and in Fig 3B, transgene #5’s GFP reporter). (TIF) [file pgen.1005889.s002.tif]
